# Stem Cell-Associated Proteins and Extracellular Matrix Composition of the Human Atrioventricular Junction

**DOI:** 10.3390/cells13242048

**Published:** 2024-12-11

**Authors:** Annika Thorsell, Linnéa Sjölin, Evelin Berger, Anders Jeppsson, Anders Oldfors, Victoria Rotter Sopasakis, Kristina Vukusic

**Affiliations:** 1 Proteomics Core Facility, Sahlgrenska Academy, University of Gothenburg, 40530 Gothenburg, Sweden; 2 Department of Laboratory Medicine, Institute of Biomedicine, Sahlgrenska Academy, University of Gothenburg, 41345 Gothenburg, Sweden; 3 Region Västra Götaland, Department of Cardiothoracic Surgery, Sahlgrenska University Hospital, 41345 Gothenburg, Sweden; 4 Department of Molecular and Clinical Medicine, Institute of Medicine, Sahlgrenska Academy, University of Gothenburg, 40530 Gothenburg, Sweden; 5 Department of Pathology, Sahlgrenska University Hospital, 41345 Gothenburg, Sweden; 6 Region Västra Götaland, Department of Clinical Chemistry, Sahlgrenska University Hospital, 41345 Gothenburg, Sweden

**Keywords:** stem cell niche, atrioventricular junction, heart, cardiac regeneration, global quantitative proteomics, mRNA-seq, cardiomyocytes, extracellular matrix

## Abstract

The human heart regenerates slowly through life, but how new cells are generated is mostly unknown. The atrioventricular junction (AVj) has been indicated as a potential stem cell niche region. Little is known about the protein composition of the human AVj. To map the extracellular matrix (ECM) and expression of stem cell-related biomarkers, this study compares protein and gene expression patterns in AVj and Left Ventricular (LV) tissues. Biopsies were collected from 15 human hearts. Global quantitative proteomics and mRNA sequencing were used to identify differentially expressed proteins and altered genes. Of the total 4904 identified proteins, 1138 were differently expressed between the AVj and LV. While the top proteins in LV were involved in cardiac motor function and energy regulation, the AVj displayed proteins associated with early cardiomyocyte development, differentiation, proliferation, migration, and hypoxia. Furthermore, several developmental signalling pathways, including TGF-β, TNF, WNT, Notch, and FGF, were represented. RNA-seq data verified that the expressed genes were involved with differentiation, cell growth, proliferation, or ECM organization. Immunohistochemistry confirmed the expression of the stem cell-related biomarkers NPPA and POSTN in the AVj, further strengthening the hypothesis of the AVj as a specialized microenvironment conducive to stem cell niche activity.

## 1. Introduction

New cardiomyocytes are generated in childhood, but the turnover decreases down to 1% per year in adults, while endothelial and mesenchymal cells show much higher cell turnover throughout life [1,2]. Where and how new cardiac cells are formed in adult human hearts is unknown. Animal models suggest the dedifferentiation of adult cardiomyocytes as the cell source for the regeneration of damaged myocardium [3,4]. We previously identified an anatomic region in adult rodent and human hearts with stem cell niche properties [5,6,7].

A stem cell niche is a reservoir of cells that can self-renew, divide, and give rise to daughter cells that migrate out of the niche and differentiate into tissue-specific cell types. Studies of tissues with rapid cell turnover, e.g., hair follicles [8], bone marrow, gonads, and small intestines [9,10], have established the concept of stem cell niches. The fundamental features are low oxygen tension (hypoxia) [11,12,13,14,15,16] and specific composition of the extracellular matrix (ECM) [17], which provide an optimal milieu for the maintenance of stem cells. For the migration of stem cells from the niches of the intervertebral disk region, it has been suggested that collagen fibres play an important role [18]. The central signalling pathways involved in the regulation of stem cell activity or quiescence include the noncanonical Wnt [19] and TGF-β/BMP2 [20], Notch [21], TNF [22], and FGF [23].

Whether a stem cell niche exists in the heart or not has been debated [24,25]. Several progenitor populations have been suggested as a potential regeneration source. This includes cells expressing embryonic cardiac stem cell markers ISL1 [26], SSEA4 [27], NKX2.5 [26], and WT1 [28]. In mice, Sca1-expressing cells have been reported to be involved in cardiac repair [29]. Small progenitor populations have been identified in different regions of human adult heart tissue as well, including MDR1+ and Cardiac Side Population cells [30,31,32,33].

Stem cell niches in the skin have been identified using DNA labelling techniques, e.g., 5-bromo-2-deoxyuridine (BrdU) [8,34], tracing slow-cycling cells that are considered as stem cells. Nests of BrdU+ cells have been identified in apex and atria in murine hearts [35].

Previously, we combined BrdU labelling with physical exercise in order to activate endogenous stem cells in a rat model. A novel potential niche was found in the atrioventricular junction (AVj), harbouring slow-cycling BrdU+ cells and cells expressing the stem cell-related biomarkers MDR1 and Sca1 [5]. Interestingly, the AVj was also previously described as the origin of proliferating cells in salamanders following cryo-injury of the ventricle [36]. To further elucidate whether a niche exists in the human AVj, explanted organ donor hearts were analysed. The human AVj, at the base of the mitral and tricuspid valves, showed features of a stem cell niche with the expression of biomarkers related to stem cells, hypoxia, proliferation, and migration [6,7].

To improve cardiac regeneration, we need to improve our understanding of the organization of cardiac stem cells and their surrounding microenvironment. The protein composition of the proposed niche region remains largely unknown. Thus, this study combines global quantitative proteomics, mRNA sequencing, and immunohistochemistry to explore the protein composition and molecular mechanisms in the AVj compared to the left ventricle (LV) in paired biopsies from explanted human hearts. Proteomics allow the study of several thousands of proteins and has the potential to uncover proteins, biological processes and pathways that influence cardiac regeneration in the AVj stem cell niche environment. mRNA-seq and immunohistochemistry analysis further strengthened our proteomics results and our hypothesis that the AVj might serve as a stem cell niche.

## 2. Materials and Methods

### 2.1. Ethics

In this study, we used explanted human hearts that were not suitable for heart transplantation. Written informed consent stating that their organs could be used for medical purposes other than organ donation was obtained from the donors via the organ donor register or next of kin. The hearts were collected for homograft procurement. Following the harvest of heart valves, the hearts were used in the present study.

### 2.2. Human Cardiac Biopsies

**Figure 1:**
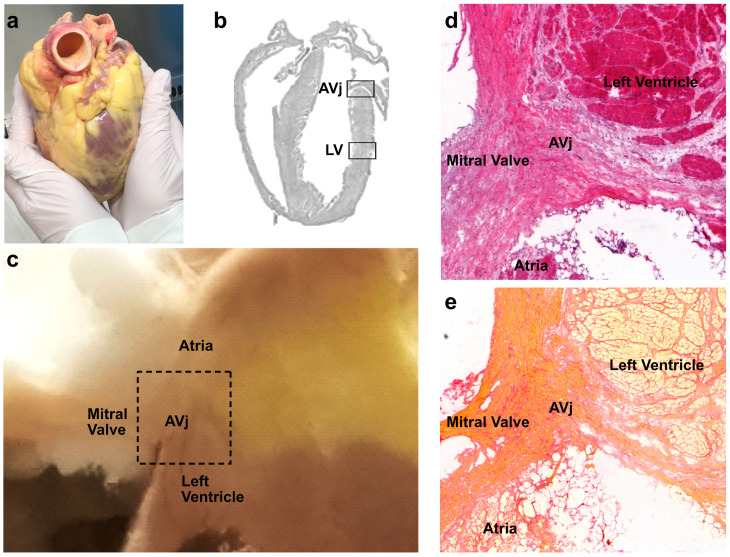
Overview of the atrioventricular junction (AVj). (**a**) Explanted human heart prior to biopsy collection. (**b**) Map over a murine heart visualizing the regions corresponding to the biopsies from the human hearts. (**c**) One representative frozen AVj biopsy, where the black dashed box shows the approximate size of the sample for global quantitative proteomics. (**d**) The histology of the AVj with the mitral valve and the surrounding left ventricle and atria regions with Haematoxylin–Eosin staining and (**e**) Picric Sirius staining showing collagen content in red.

Paired cardiac biopsies from the lateral side of the hearts were obtained from 15 multi-organ donors (aged 19–75 years). The first biopsy site was the proposed stem cell niche region AVj, and the second was the middle of the LV (Figure 1b). The biopsies from 6 donors used for proteomics (Figure 1c) were fresh-frozen in liquid nitrogen and stored at −80 °C until further analysis. For RNA sequencing, histology, and immunohistochemistry, see below. The clinical background of the included multi-organ donors is summarized in Table 1.

### 2.3. Histology

Biopsies from the AVj and LV were embedded in Tragacanth mounting medium (Histolab Products AB, Gothenburg, Sweden), snap-frozen in liquid nitrogen, and stored at −80 °C. The frozen tissue from six donors was sectioned into 7 μm serial sections and stained with Haematoxylin–Eosin, Picric Sirius red, or used for immunohistochemistry.

### 2.4. Proteomic Sample Preparation

Relative protein quantification was performed to compare the protein expression in the AVjs and LVs from the donors. Proteins were extracted in lysis buffer (2% sodium dodecyl sulphate, 50 mM triethylammonium bicarbonate) using a FastPrep^®^-24 instrument (Matrix D, MP Biomedicals, SantaAna, CA, USA). Protein concentrations were determined using Pierce BCA Protein Assay Kit (Thermo Fisher Scientific, Waltham, MA, USA) on a Benchmark Plus microplate reader (BIO-RAD, Hercules, CA, USA). A representative reference pool including tissue from all samples was prepared. Aliquots (30 µg) from the samples and the reference pool were processed by the modified filter-aided sample preparation method [34]. In short, the samples were transferred to Microcon-Biomax membrane 30kDa Centrifugal Filter Units (Merck, Rahway, NJ, USA), reduced (dithiothreitol 100 mM, 56 °C for 30 min), and washed several times with urea (8 M) and once with digestion buffer (DB, 50 mM HEPES, 0.5% sodium deoxycholate (SDC)) prior to alkylation (methyl methanethiosulfonate, 10 mM, 30 min in room temperature). The samples were digested with trypsin (Pierce MS grade Trypsin, Thermo Fisher Scientific, Waltham, MA, USA, ratio 1:100) at 37 °C overnight, and then an additional portion of trypsin was added and incubated for another three hours. Peptides were collected by centrifugation and labelled using tandem mass tag (TMT) 11-plex isobaric mass tagging reagents (Thermo Fisher Scientific, Waltham, MA, USA), according to the manufacturer’s instructions. The labelled samples and reference were combined into one pooled sample per set, and SDC was removed by acidification with 10% trifluoroacetic acid. The TMT sets were purified using Pierce peptide desalting spin columns (Thermo Fisher Scientific, Waltham, MA, USA) according to the manufacturer’s instructions prior to basic reversed-phase liquid chromatography fractionation. Peptide separation was performed using a Dionex Ultimate 3000 UPLC system (Thermo Fischer Scientific, Waltham, MA, USA) and a reversed-phase XBridge BEH C18 column (3.5 μm, 3.0 × 150 mm, Waters Corporation, Milford, MA, USA) with a gradient from 3% to 90% acetonitrile in 10 mM ammonium formate at pH 10.00 over 30 min at a flow of 400 µL/min. The 40 fractions were concatenated into 20 fractions and dried and reconstituted in 3% acetonitrile and 0.1% trifluoroacetic acid.

### 2.5. Nano-Liquid Chromatography and Mass Spectrometry Analysis

The fractions were analysed on an Orbitrap Fusion Tribrid mass spectrometer interfaced with an Easy-nLC1200 liquid chromatography system (all Thermo Fisher Scientific). Peptides were trapped on an Acclaim Pepmap 100 C18 trap column (100 μm × 2 cm, particle size 5 μm, Thermo Fisher Scientific) and separated on an in-house packed analytical column (30 cm × 75 μm, particle size 3 μm, Reprosil-Pur C18, Dr. Maisch, Ammerbuch-Entringen, Germany) using a stepped gradient from 5% to 35% acetonitrile in 0.2% formic acid over 77 min at a flow of 300 nL/min. Precursor ion mass spectra were acquired at a resolution of 120,000 and an *m*/*z* range of 380–1380. Using a cycle time of 3 s, the most abundant precursors with charges 2–7 were isolated with an *m*/*z* window of 0.7 and fragmented by collision-induced dissociation at 35%. Fragment spectra were recorded in the ion trap at the Turbo scan rate. Dynamic exclusion was set to 60 sec. The five most abundant MS2 fragment ions were isolated using multi-notch isolation for further MS3 fragmentation. MS3 fragmentation was performed using higher-energy collision dissociation at 65%, and the MS3 spectra were recorded in the Orbitrap at 50,000 resolution and an *m*/*z* range of 100–500.

### 2.6. Database Matching and Protein Quantification

Raw files were processed and analysed with Proteome Discoverer (version 3.0, Thermo Fisher Scientific, Waltham, MA, USA). The data were matched against the Homo sapiens UniProtKB SwissProt database (20,422 reviewed entries, April 2023) using Sequest as a search engine with a precursor tolerance of 5 ppm and a fragment ion tolerance of 0.6 Da. Tryptic peptides were accepted with 1 missed cleavage. Methionine oxidation was set as a variable modification, and cysteine alkylation, TMT on lysine, and peptide N-termini were set as fixed modifications. A percolator was used for peptide spectra match (PSM) validation with a strict false discovery rate (FDR) threshold of 1%. For quantification, TMT reporter ions were identified in the MS3 higher-energy collision-induced dissociation spectra with 3 mmu mass tolerance. The TMT reporter intensity values for each sample were normalized on the total peptide amount. The synchronous precursor selection threshold was set to 65%, and a Sequest HT threshold score of 2 was chosen. Only unique peptides were used for relative quantification, and proteins were required to pass a protein FDR of 5%.

### 2.7. Processing of Quantitative Proteomics Data

Statistical and bioinformatics analyses of the proteomic data were performed with Perseus software [37] (version 2.0.11). Gene Ontology (GO) annotations were downloaded from the UniProt database (November 2023). To identify differentially expressed proteins (DEP), a paired Welch’s *t*-test on log2-transformed data followed by Benjamini–Hochberg correction for multiple testing (FDR 0.05) was conducted. Proteins with an FDR < 0.05 and fold change (FC) ≥ 50% (log2 FC ≤ −0.58 or ≥0.58) were considered as differentially expressed. A principal component analysis (PCA) and visualization of DEPs in a volcano plot were performed on all proteins. Heatmaps and hierarchical clustering were generated for DEPs with selected GO terms using Pearson correlation distances in Perseus. A gene set enrichment analysis (GSEA) was performed separately on the upregulated and downregulated DEPs using Panther (20240807 release). Significant and relevant biological processes were visualized using the Seaborn package (v0.13.2) for Python (v 3.9.18).

The prediction of protein–protein interactions was performed using STRING (v.12.0) [38] and the Markov clustering algorithm to construct protein–protein interaction networks based on all DEPs and proteins showing upregulated expression in each respective group.

### 2.8. RNA Extraction

Tissue for gene analyses was placed in RNAlater (Qiagen, Valencia, CA, USA) and stored at −80 °C. A total of 30–100 mg of tissue per sample was homogenized in a TissueLyser LT and lysed with Qiazol (Qiagen). The RNA was purified with the RNeasy Mini Kit in the QIAcube (Qiagen) according to the manufacturer’s protocol. DNase1 was used to remove genomic DNA.

### 2.9. RNA Sequencing Analysis

Library construction was performed using Illumina Truseq stranded total RNA with the Illumina Ribozero method. Clustering was performed by ‘cBot’, and samples were sequenced on NovaSeq6000 (NovaSeq Control Software 1.6.0/RTA v3.4.4) with a 2 × 51 setup using ‘NovaSeqXp’ workflow in ‘S1’ mode flow cell. The Bcl to FastQ conversion was performed using bcl2fastq_v2.19.1.403 from the CASAVA software suite. The processing of FASTQ files was carried out by the SciLifeLabNationalGenomics Infrastructure at the Uppsala Multidisciplinary Center for Advanced Computational Science, Sweden. Sequenced reads were quality-controlled with the FastQC software and pre-processed with Trim Galore.

### 2.10. Statistics and Bioinformatics for Gene Analysis

The raw data were aligned with the human GRCh38.107 reference library from the Ensembl genome browser (https://www.ensembl.org/Homo_sapiens/Info/Index (accessed on 30 August 2022)), and the resulting BAM files were used for bioinformatics analysis. Statistical and bioinformatics analyses of the gene data were performed with Qlucore Omics Explorer 3.9 (Qlucore AB, Lund, Sweden). Qlucore Omics Explorer software v3.9 utilizes trimmed mean of M-values normalization and genomic feature length normalization and performs log2 transformation of the data prior to analysis. Transcripts with counts ≥ 5 were included in the analysis. A paired group comparison of seven individuals was performed, and levels of significance for differences between group means were determined with a *t*-test. An FDR < 0.05 was considered significant.

### 2.11. Immunohistochemistry

Frozen tissue sections of human AVjs and LVs were fixed in acetone for 10 min at −20 °C and thereafter washed with phosphate-buffered saline (PBS). The blocking of background was performed with 1% bovine serum albumin (BSA) and 5% goat serum (Invitrogen) in PBS for 30 min. Primary antibodies for NPPA (AB209232, Abcam, ratio 1:1000) and POSTN (MA5-27179, Thermo Fisher Scientific, ratio 1:1000) were added to the sections and left to incubate at 4 °C overnight in a moisture chamber. After washing with PBS, secondary antibodies of goat anti-rabbit, Alexa 647 (A21206, Thermo Scientific, ratio 1:1000), and goat anti-mouse, Alexa 546 (A11030, Invitrogen, ratio 1:1000), were added to the samples for 1h at room temperature (RT). After washing with PBS, a Zenon kit (Invitrogen) was used to directly conjugate Alexa 488 fluorochrome with cTnT antibody (MS-295-P1, Thermo Fisher Scientific, ratio 1:50), which was added to the sections for 1h at RT. The sections were thereafter washed with PBS and then fixed with Histofix (Histolabs) for 15 min. Following a final wash with PBS, the sections were mounted using prolong gold antifade reagent with nuclei staining 4′,6-diamidino-2-phenylindole (DAPI) (Invitrogen).

### 2.12. Bioimage Analysis

The sections were imaged with an ECLIPSE Ti-E inverted microscope (Nikon Corporation) and a Zyla sCMOS VC884 camera (Andor). Large field images were acquired by stitching together 5 × 5 photos, taken at 20× at three different z-levels. The images were analysed using ImageJ (v1.54f, Fiji distribution). Thresholds for each colour channel were set globally across all images, using the negative controls to find levels with a minimum background signal.

## 3. Results

### 3.1. Histology of the AVj

Paired biopsies from the explanted human hearts were taken from the AVj and LV (Figure 1a–c) and stained with Haematoxylin–Eosin and Picric Sirius red for histology (Figure 1d,e). The biopsies of the AVj contained a small part of the left atria, the left ventricle, the mitral valve, and epicardial adipose tissue. Histology of the myocardium in the AVj displayed the cardiomyocytes near the mitral valve. The cardiomyocytes closest to the valve were small, but the size increased further into the atria and ventricle. The Picric Sirius red staining revealed that the connective tissue between the myocardium and the mitral valve was rich in collagen, with some collagen strands stretching into the myocardium of both the atria and the ventricle.

### 3.2. Differences Between the AVj and LV with Global Quantitative Proteomics

**Figure 2:**
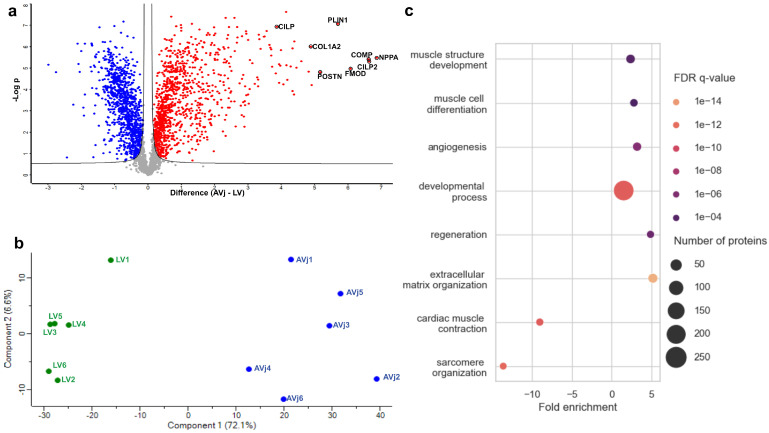
Overview of the results found in the statistical analyses of the proteomic data. (**a**) Volcano plot of *p*-values vs. log_2_ FC in the AVj region compared with the LV region. Significantly upregulated and downregulated proteins in the AVj are highlighted in red and blue, respectively (*p* ≤ 0.05, s0 = 0.1). Selected proteins among the most enriched in the AVj region are highlighted and labelled. (**b**) PCA of the AVj (blue) and the LV (green) based on their proteomic expression profiles. The first and second components segregate the heart areas and account for 72.1 and 6.6% of the variability, respectively. (**c**) Enrichment analysis of the Gene Ontology biological processes displayed as a dot plot of enriched biological processes derived from the 1133 proteins displaying significantly altered expression patterns (log2 fold change ≥ 0.5, FDR q-value ≤ 0.05). The colour of the dot designates the significance of the change in expression (adjusted *p*). The size of the dot signifies the number of enriched proteins.

To obtain a high purity of cells from the AVj region while preventing contamination from LV or LA cells, the biopsies collected for proteomic analyses were much smaller, approximately 3 × 3 mm, as outlined in Figure 1c (N = 6, Table 1). Global quantitative proteomics analysis identified a total of 27,598 sequence peptides corresponding to 4904 proteins. Among these, 1138 DEPs were identified in the comparison between the AVj and LV regions (Log2 FC = 0.58, FDR < 0.05, Table S1). The DEPs comparing the two groups are visualized in a volcano plot (Figure 2a). A total of 590 proteins showed significantly higher expression levels in the AVj and 548 in the LV region. The large number of DEPs demonstrated substantial differences in protein expression between the two regions. Many DEPs had high sequence coverage and were quantified with a high number of unique peptides and peptide spectral matches (PSMs), reflecting the high quality of their quantification in human tissue samples. The PSM is the number of times peptides from a specific protein are selected for fragmentation (MS/MS), which is used for protein identification and quantification. A high PSM relative to the number of peptides can potentially indicate high protein abundance. Additionally, global quantification is biased toward proteins with molecular weights higher than 20 kDa, as the number of tryptic peptides used for quantification depends on the protein sequence length. Consequently, proteins with lower molecular weights tend to have fewer peptides and PSMs than those with higher molecular weights. Therefore, the identification of low-molecular-weight DEPs may suggest they are highly abundant in the tissue as well as strongly regulated (high Log2 fold change, Table S1).

To provide a comprehensive overview of the similarities and differences in the proteome of the two regions, we employed PCA for the quantified proteins, demonstrating a clear separation of the two regions (Figure 2b). Most of the data variability was mainly explained by component 1 (72%), which stratified the samples by anatomical region. Several primary contributors to PC1 and the segregation of the anatomical regions were mainly driven by upregulated proteins in the AVj, e.g., ECM-associated proteins such as CILP2, COMP, FMOD, BGN, and COL1A, as well as the stem cell-related markers NPPA and POSTN. Typical DEPs in the LV region were proteins involved in mitochondrial processes, energy regulation, and cardiac muscle contraction, such as MT-ND2, MT-ND5, MT-CO1, MT-ATP6, MYL1, and MYL3. Table 2 and Table 3 list the top 25 most regulated proteins in either the AVj or LV region, as well as their biological functions. The MS data together, with the low FDR and high fold change, indicate a high quality of the quantification but also indicate a high abundance of the topmost regulated DEPs.

GO enrichment analysis was performed on the DEPs to identify which biological processes were over-represented in the two cardiac regions. Several GO terms related to muscle development, angiogenesis, regeneration, and ECM organization were enriched in the AVj (Figure 2c). Importantly, among the downregulated biological processes in the AVj were proteins related to more mature cardiomyocyte functions, such as sarcomere organization and cardiac muscle contraction.

### 3.3. Gene Expression in the AVj Compared to the LV

**Figure 3:**
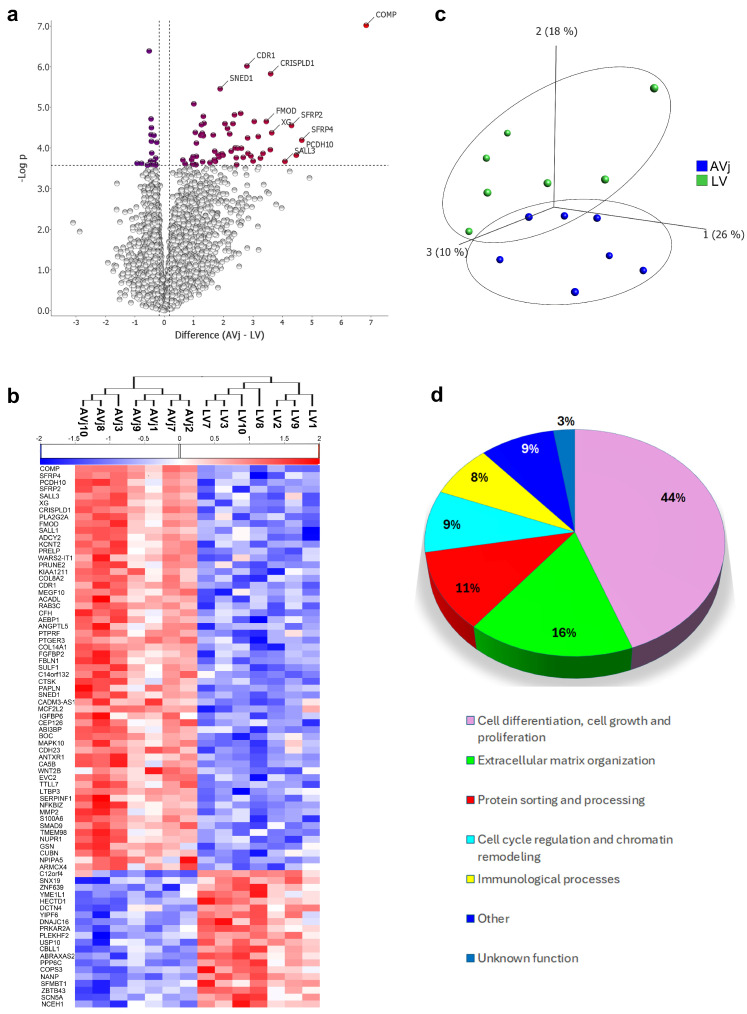
Gene expression analysis of AVj and LV was performed using RNA sequencing, followed by paired group comparison of seven individuals. (**a**) Volcano plot displaying the difference in gene expression between the AVj region and the LV (log2 fold change, FDR q-value < 0.05). (**b**) Heatmap between the AVj and LV regions showing significantly altered transcripts (log2 fold change, FDR q-value < 0.05). (**c**) PCA score plot (first three components) of the total number of transcripts detected, showing a separation between the AVj (blue) and left ventricle (green) with regard to gene expression. (**d**) Pie diagram showing the proportion of transcript categories for the significantly altered transcripts in the AVj compared to the LV.

RNA was isolated from the AVj and LV of the hearts (N = 7, Table 1), sequenced, and analysed. The samples were paired for RNA isolated from the different regions of the same individual. In total, 14,460 transcripts were detected in the gene data set, 79 of which showed significantly altered expression when comparing the AVj to the LV (FDR < 0.05), (Figure 3a,b). Using PCA, a separation with regard to gene expression could be seen between the AVj and LV (Figure 3c). Of the 79 significantly different transcripts between the regions, the majority of the transcripts were of genes associated with differentiation, cell growth, and proliferation (44%) (Figure 3d), e.g., *COMP*, *SFRP2* and 4, *SALL1* and 3, and *CRISPLD1* (Figure 3b), followed by genes involved in extracellular matrix organization (16%), protein sorting and processing (11%), cell cycle regulation and chromatin remodelling (9%), and immunological processes (8%) (Figure 3d).

### 3.4. Comparison of Proteomics and Gene Expression Data

A comparison between the altered genes and DEPs demonstrated that 25% of the genes that showed significant alterations in expression also had similar changes in protein levels, e.g., COMP, PLA2G2A, FMOD, and PRELP (Table 4). The overlap points to genes and proteins that are tightly regulated at both the transcript and protein levels. The majority of the overlapping genes aligned with processes involved in cell differentiation and extracellular matrix organization. With both techniques, the enriched biological processes in the AVj region were associated with stem cells and stem cell niches.

### 3.5. Stem Cell Niche-Related Proteome of the AVj

**Figure 4:**
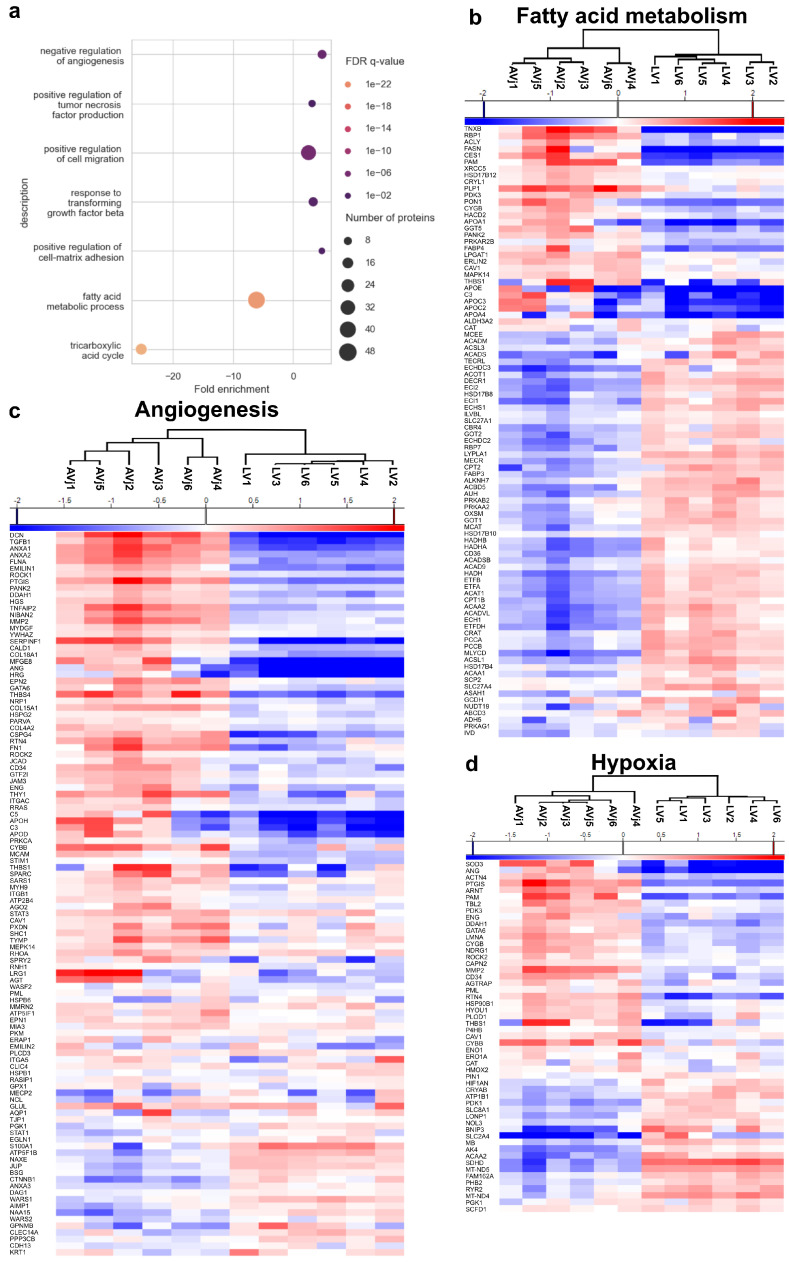
Selected biological processes from the proteomics analysis that were enriched in the AVj. (**a**) Selected enriched biological processes in the AVj related to stem cell niche biology. The size of the spheres shows the number of proteins involved in the specific biological process. The colour of the spheres shows the FDR q-value. (**b**–**d**) Heatmaps over differentially expressed proteins related to stem cell niche biology: fatty acid metabolism, angiogenesis, and hypoxia. The red and blue colour gradients represent the relative increase or decrease in protein expression levels, respectively.

The more detailed and specific GSEA of the proteomics data revealed that proteins involved in stem cell niche processes are enriched in the AVj. These were processes such as positive regulation of cell migration, positive regulation of the TNF pathway, response to the TGF-β pathway, and ECM adhesion (Figure 4a). In addition, when investigating the sub-terms of the GO term for angiogenesis, we found that the negative regulation of angiogenesis was the most significantly enriched one. Additionally, there was an observed significant reduction in fatty acid metabolism and the tricarboxylic acid cycle, both important in the mitochondrial processes and energy regulation in the LV region.

Heatmaps of the DEPs associated with angiogenesis, fatty acid metabolism, and hypoxia revealed a separation between the AVj and LV (Figure 4b–d). The unsupervised hierarchical clustering demonstrated two distinct clusters in all heatmaps.

### 3.6. Enrichment of Developmental Signalling Pathways in the AVj

**Figure 5:**
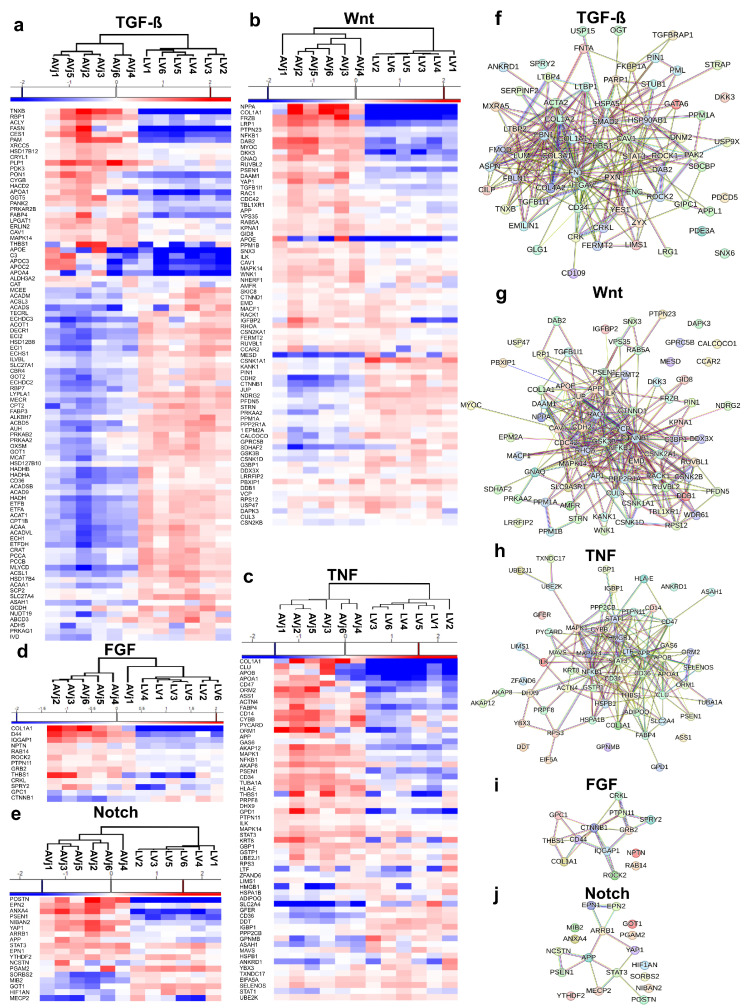
Stem cell niche-related signalling pathways from the proteomics analysis that were enriched in the AVj. (**a**–**e**) Heatmaps of differentially expressed proteins involved in the TGF-β, Wnt, TNF, FGF, and Notch pathways. The red and blue colour gradients represent the relative increase and decrease in protein levels, respectively. (**f**–**j**) Protein–protein interaction network analyses using the STRING database showing the interactions between the differentially expressed proteins involved in the TGF-β, Wnt, TNF, FGF, and Notch pathways. The lines between the nodes are coloured according to the type of interaction. The cyan and purple lines are known interactions from curated databases and experimentally determined interactions, respectively. The green, red, and dark blue lines are predicted interactions based on gene neighbourhood, gene fusion, and gene co-occurrence, respectively. The yellow, black, and light blue lines are other interactions based on textmining, co-expression, and protein homology, respectively.

Most of the proteins involved in the TGF-β, Wnt, TNF, FGF, and Notch signalling pathways showed a clear separation between the two anatomical regions (Figure 5a–e). These pathways are involved in embryonal development, cell differentiation, regeneration, and proliferation and are thus central in stem cell niche biology. Since these signalling pathways are large and include a high number of proteins, we verified that the DEPs for the different pathways interact with each other by using the STRING protein interaction database. The results showed tight clusters including the majority of DEPs related to these pathways and confirmed that they interact with each other (Figure 5f–j).

### 3.7. Extra Cellular Matrix Composition and Immunohistology of the AVj

**Figure 6:**
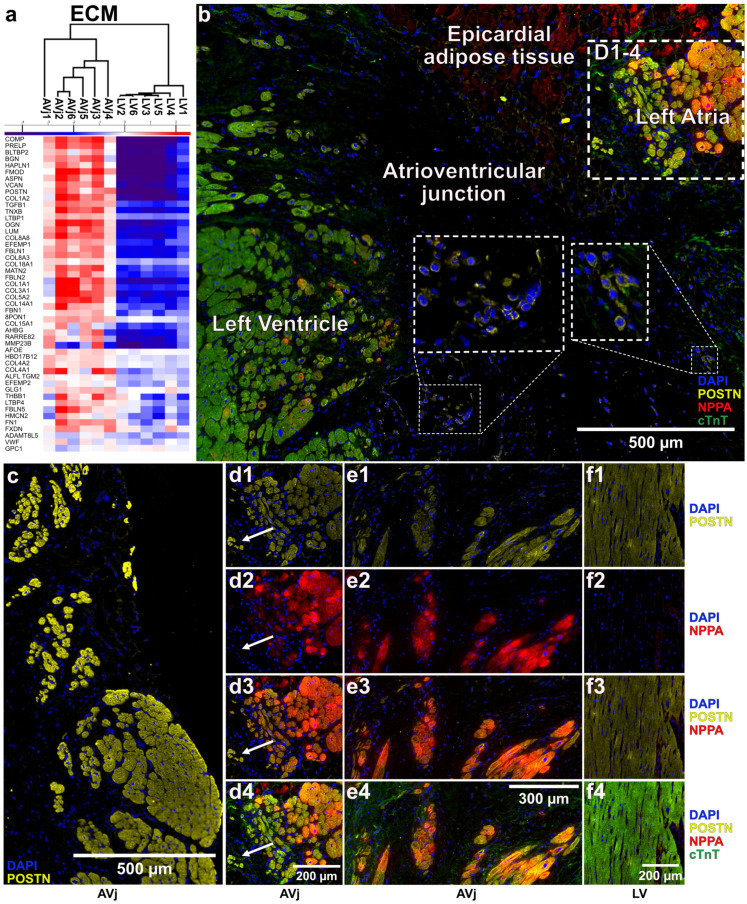
Extracellular matrix (ECM) proteins enriched in the AVj and immunohistological (IHC) verification of proteomic data. (**a**) Heatmap of differentially expressed ECM proteins. The red, blue, and purple colour gradients represent the relative increase or decrease in protein expression levels, with red corresponding to enrichment in the AVj, blue with reduction, and purple with a further reduction. (**b**) Immunohistochemistry (IHC) of the human heart tissue sections showing an overview of the AVj region. Periostin (POSTN) is shown in yellow, Natriuretic peptide A (NPPA) in red, cardiac troponin T (cTnT) in green, and cell nuclei stained with DAPI in blue. A subpopulation of POSTN-positive cells that were negative for cTnT is magnified. (**c**) IHC of cardiomyocytes in the AVj of a human heart. POSTN is shown in yellow, and nuclei stained with DAPI are in blue. (**d1**–**f4**) IHC of tissue from three human hearts showing cardiomyocytes stained with POSTN in yellow (**d1**,**e1**,**f1**) and NPPA in red (**d2**,**e2**,**f2**). Both stainings are shown overlayed with each other (**d3**,**e3**,**f3**). A cluster of cells positive for POSTN but negative for NPPA in (**d3**) is pointed out with an arrow. POSTN and NPPA were overlayed with cTnT, shown in green (**d4**,**e4**,**f4**). All images also show nuclei stained in blue. The images in (**d1**–**d4**) and (**e1**–**e4**) are from the AVj, and the images in (**f1**–**f4**) are from the left ventricle.

Since the composition of the ECM is of importance to keep stem cells in an undifferentiated state, we mapped the composition of the ECM in the AVj using proteomics data (Figure 6a). The heatmap over ECM-related DEPs showed a very strong and clear difference in the composition and an upregulation in the AVj. The most upregulated proteins in the AVj region were NPPA, POSTN, COMP, PRELP, BGN, FMOD, FRZB, and COLL1A2, all known to be associated with stem cells.

The proteomic findings were verified using IHC. Sections of human cardiac AVj and LV tissue were stained for the stem cell-related markers NPPA and POSTN, which were among the most enriched proteins in the AVj. Additionally, the sections were stained with DAPI to show the cell nuclei, and for cardiac troponin T (cTnT), a cardiac sarcomere component, to discern cardiomyocytes from the other cardiac cell types (Figure 6(b–f4)). POSTN was highly expressed in the smallest cardiomyocytes in the AVj, closest to the mitral valve (Figure 6b,c). Interestingly, there was a subpopulation of smaller POSTN-expressing cardiomyocytes that did not show any staining for cTnT (Figure 6b. Overall, low expression of POSTN was detected in all cardiomyocytes, both in the AVj and LV (Figure 6(d1,e1,f1)). However, more intense staining of POSTN was observed in small cardiomyocytes at the edge of the myocardium in the AVj (Figure 6c). NPPA was mainly found in and around multiple clusters of cardiomyocytes in the myocardium of the AVj (Figure 6(d2,e2)) and was not detected at all in the LV (Figure 6(f2,f4)). Several cardiomyocytes showed co-expression of POSTN and NPPA (Figure 6(d3,e3,f3)), as well as with cTnT (Figure 6(d4,e4)). However, there were also several cardiomyocytes in the AVj where NPPA expression was not observed, such as the right side of Figure 6b and the cells pointed out with an arrow in Figure 6(d3).

## 4. Discussion

The molecular mechanisms of the human heart are particularly difficult to study due to the lack of access to non-diseased human heart tissue. Most of the studies on cardiac regeneration have been performed on animal models. Although providing very important information, animal models are not always equivalent to humans [3,4,39,40]. In this study, we used paired human biopsies from multi-organ donors and compared global protein and gene expression between the previously proposed stem cell niche region AVj and the myocardium of LV. The chemical labelling with TMT utilized in quantitative protein analysis enabled sample multiplexing, reducing technical variability to below 10%. Additionally, MS3-based quantification with synchronous precursor selection (SPS) minimizes peptide interference and enhances quantitative sensitivity and accuracy [41,42,43]. High-quality protein quantification was also demonstrated in the present study by the large number of unique peptides and high PSM counts used in the quantification. Furthermore, the substantial number of DEPs reflected significant protein expression differences between the two regions. This was expected, as the AVj is the collagen-rich annulus fibrosus of the mitral valve, while the LV is muscle tissue dominated by sarcomeric muscle contraction proteins, as can be seen in Figure 1e. It is important to note that TMT ratio distortions can lead to ratio compression, where experimentally measured fold changes are smaller than the actual differences between samples, especially for proteins with relatively large fold changes [44,45]. Therefore, proteins with a log2 FC > 3–4 in the AVj may be considered undetected or very low in abundance in the LV.

The top proteins in the LV were involved in cardiac motor function and energy regulation. For example, MYL1 and MYL3 are components of myofibrils, ANKRD2 is involved in the stretch response of muscles, MT-ND2, MT-ND5, MT-CO1, SDHD, and SDHC are part of the mitochondrial respiratory chain, and SLC2A4 is a glucose transporter (Table 3).

Among the top proteins of the AVj, there were many that were associated with stem cells, development, and regeneration. ECM proteins are known to be critical for maintaining stem cell niches, and the most enriched were COMP, CILP2, NPPA, POSTN, PRELP, BGN, FMOD, FRZB, and COLL1A2 (Table 2). Both NPPA and FRZB are involved in embryonic cardiac development and are members of the Wnt signalling pathway [46,47]. POSTN has been reported to be expressed by skeletal stem cells and regulated by the hypoxia-related Hif1-α [13,48]. BGN is involved in the regulation of the differentiation of tendon stem cells [49]. Furthermore, BGN is required for the stable collagen matrix formation of infarct scars and for the preservation of cardiac hemodynamic function [50]. The CILP2 protein, also known as osteoglycin, attenuates cardiac fibrosis by suppressing cardiac myofibroblast proliferation [51]. Taken together, these are all known to be related to stem cell biology in different tissues.

GSEA suggested that many enriched processes in the AVj are related to an immature, developing cardiac phenotype, such as muscle cell differentiation and muscle structure development, as well as a reduction in processes related to mature cardiac function, such as cardiac muscle contraction and sarcomere organization (Figure 2c).

Several biological processes also indicate a stem cell niche environment. The negative regulation of angiogenesis by upregulated proteins, such as DCS, SERPINF1, THBS4, APOH, and HRG, promotes a hypoxic environment. This is in line with our previous findings of nuclear Hif1-α expression and less abundant endothelial CD31 cells in the human AVj, suggesting a hypoxic niche in the human heart [6,7,52].

GSEA revealed that proteins involved in stem cell niche processes, such as migration, were enriched in the AVj (Figure 3a). One of the enriched pathways was the reduction in fatty acid metabolism, which is a step towards postnatal maturation of cardiomyocytes since the shift from glycolysis to fatty acid oxidation marks an exit from the cell cycle and loss of cardiac regeneration [53]. Additionally, there was a reduction in the tricarboxylic acid cycle, which was described as a regulator of cell pluripotency [54]. A large number of the upregulated proteins are involved in several developmental signalling pathways, including TGF-β, TNF, Wnt, Notch, and FGF, which are well-known drivers of cell differentiation, migration, and proliferation, as reviewed in [55,56,57,58,59,60,61,62].

Protein–protein interaction and functional enrichment analysis using STRING also revealed tightly clustered proteins within these pathways, indicating the biological relevance of these proteins (Figure 5) and supporting our hypothesis of a stem cell niche in the AVj region.

Interestingly, in the heatmap for the FGF-related proteins (Figure 5d), the AVj sample from donor 1 is grouped together with the LV samples rather than the other AVj samples. That sample is also notably distinct in the PCA plot (Figure 2b) compared to the rest of the donors. This might be explained by the higher age and clinical background of this donor.

The enrichment of migration-associated proteins, alongside those involved in developmental signalling pathways, is consistent with the previously suggested model of a hypoxic stem cell niche environment in the human AVj. Further functional studies following the progeny of the cells in the AVj are required to test the hypothesis that In the AVj, the immature cardiac cells reside, anchored to the ECM, producing daughter cells that migrate out of the niche environment.

The gene expression data also confirmed the separation between the AVj and LV, showing that most of the transcripts in the AVj were associated with differentiation, cell growth, proliferation, and the ECM (Figure 3).

A limitation of the present study is the lack of a strong correlation between the transcriptomics and proteomics data, with only a 25% overlap. A limited correlation between proteomics and transcriptomics arises because mRNA levels do not always directly predict protein abundance. This can be attributed to factors such as post-transcriptional regulation, translational efficiency, protein degradation, and post-translational modifications, as well as technical differences [63]. Despite this, combining RNA-seq and proteomics provides a more complete view of gene expression and function.

Importantly, both the proteomics and transcriptomics results independently highlighted an increase in processes associated with differentiation, cell cycle regulation, proliferation, and ECM regulation in the AVj. The correlated proteins upregulated in the AVj were COMP, FMOD, PRELP, and PLA2G2A. Li et al. reported that PRELP promotes osteoblast differentiation through β-catenin signalling [64]. Interestingly, PLA2G2A plays a stem cell regulator role in the intestinal crypt [65] and is found at a high level in the tumour microenvironment, stimulating cancer progression and metastasis [66]. Taken together, the results from the GSEA of the RNAseq data generally matched those of the proteomics data.

The immunohistochemistry results verified the expression of two of the top proteins in the AVj that were identified with proteomics analysis. NPPA, which was the DEP with the highest FC between the AVj and LV, was detected in the cardiomyocytes in the AVj and was not expressed in the LV. POSTN, on the other hand, was expressed in cardiomyocytes in both the AVj and LV, but the intensity of the staining was strongest in smaller cardiomyocytes at the edges of the myocardium of the AVj (Figure 6). The same expression pattern was previously reported for BrdU+ cells in rat hearts [5] and the embryonic cardiac stem cell marker SSEA4 in the human AVj [6,7]. There, the expression was strong in cardiomyocytes at the myocardium border and faded out further into the LV. Additionally, in the same region, small cardiomyocytes were positive for cardiomyocyte nuclei marker PCM1 but negative for cTnT [7]. This result is reminiscent of the small cells that were positive for POSTN but negative for cTnT (Figure 6b). We interpret these small myocytes found in the AVj, with NPPA+/cTnT+ and POSTN+/cTnT+, as well as the previously reported Isl+/cTnT+, WT1+/cTnT+, SSEA4+/cTnT+, and PCM1+/cTnT- molecular signatures, as cardiomyocyte progenitors at different developmental stages.

Our findings provide new insights into the molecular differences between the AVj and the LV. The identification of proteins and genes specific for the AVj associated with early cardiomyocyte development, cell migration, and hypoxia suggests that this region might play an important role in cardiac regeneration. Future functional studies are needed to investigate the characteristics of the cells in the AVj and their potential role in cardiac regeneration. In conclusion, these distinct molecular characteristics identified with three different methods provide new evidence that the AVj may function as a potential cardiac stem cell niche. This could have implications for cardiac regeneration strategies, as understanding this niche may offer therapeutic insights into how to enhance the heart’s regenerative capacity.

## Figures and Tables

**Table 1:** Clinical background of the included multi-organ donors.

Donor	Sex	Age	Cause of Death	Other Diseases	Tissue Used for
1	F	63	Ischemic cerebral oedema, due to cardiac arrest	Ischemic heart disease, hypertension, obesity, hypothyroidism, diabetes type 2, renal insufficiency, emphysema	Proteomics, RNAseq
2	F	19	Ischemic cerebral oedema, due to cardiac arrest	Anorexia	Proteomics, RNAseq; IHC
3	F	43	Ischemic cerebral oedema, due to cardiac arrest	None	Proteomics, RNAseq
4	M	19	Ischemic cerebral oedema, due to cardiac arrest	HF in the acute setting	Proteomics
5	M	21	Ischemic cerebral oedema, due to cardiac arrest	HF in the acute setting	Proteomics
6	M	46	Ischemic cerebral oedema, due to cardiac arrest	None	Proteomics
7	F	42	Intracerebral haemorrhage	Takotsubo cardiomyopathy in the acute setting	RNAseq
8	M	52	Cardiac arrest	HF in the acute setting	RNAseq
9	F	75	Intracerebral haemorrhage	Atrial fibrillation, ischemic heart disease, previous AMI	RNAseq
10	M	74	Intracerebral haemorrhage	Previous stroke	RNAseq
11	M	63	Traumatic brain injury	Hypertension	IHC
12	F	75	Cerebral haemorrhage	Atrial fibrillation, previous AMI	IHC
13	M	54	Cardiac arrest	Suspected LAD dissection	IHC
14	M	69	Traffic accident	None	IHC
15	F	53	Cardiac arrest	None	IHC

Table 1 lists the clinical background of the organ donors. F = Female, M = Male, HF = Heart Failure, AMI = Acute Myocardial Infarction, LAD = left anterior descending artery, Proteomics = global quantitative proteomics, RNAseq = RNA sequencing, IHC = immunohistochemistry.

**Table 2:** Top 25 upregulated proteins of the AVj.

Gene ID	Accession	Protein Description	Log2 FC	FDR	Biological Function or Pathways *
NPPA	P01160	Natriuretic peptide A	6.86	3.22 × 10^−3^	Cardiac system development, stem cell marker, Wnt signalling
CILP2	Q8IUL8	Cartilage intermediate layer protein 2	6.63	3.30 × 10^−3^	ECM organization
COMP	P49747	Cartilage oligomeric matrix protein	6.61	2.61 × 10^−3^	ECM component, developmental processes
FMOD	Q06828	Fibromodulin	6.08	4.56 × 10^−3^	TGF-β signalling
PLIN1	O60240	Perilipin-1	5.69	1.13 × 10^−3^	Lipid metabolism
POSTN	Q15063	Periostin	5.16	2.38 × 10^−3^	ECM organization, developmental processes, stem cell marker
BGN	P21810	Biglycan	4.91	6.23 × 10^−3^	Cartilage development, developmental processes
COL1A2	P08123	Collagen alpha-2(I) chain	4.89	1.77 × 10^−3^	TGF-β signalling, developmental processes
HAPLN1	P10915	Hyaluronan and proteoglycan link protein 1	4.81	3.84 × 10^−3^	ECM component, developmental processes
PRELP	P51888	Prolargin	4.60	3.11 × 10^−3^	ECM component, developmental processes
ASPN	Q9BXN1	Asporin	4.45	3.48 × 10^−3^	ECM component, TGF-β signalling
ABI3BP	Q7Z7G0	Target of Nesh-SH3	4.39	2.02 × 10^−3^	ECM organization
MAMDC2	Q7Z304	MAM domain-containing protein 2	4.38	4.70 × 10^−3^	ECM component, developmental processes
OGN	P20774	Mimecan	4.33	2.38 × 10^−3^	Cartilage development, ECM component
PLA2G2A	P14555	Phospholipase A2	4.33	1.13 × 10^−3^	Inflammatory response, stem cell homeostasis
FRZB	Q92765	Secreted frizzled-related protein 3	4.28	3.39 × 10^−3^	Developmental processes, Wnt signalling
MYL7	Q01449	Myosin regulatory light chain 2	4.18	2.74 × 10^−3^	Cardiac muscle tissue development
COL1A1	P02452	Collagen alpha-1(I) chain	4.15	3.15 × 10^−3^	Cartilage development, ECM organization, Wnt signalling
MYOZ1	Q9NP98	Myozenin-1	4.14	1.13 × 10^−3^	Muscle tissue development
PCOLCE2	Q9UKZ9	Procollagen C-endopeptidase enhancer 2	4.06	4.21 × 10^−3^	Response to leukaemia inhibitory factor
COL12A1	Q99715	Collagen alpha-1(XII) chain	3.98	2.21 × 10^−3^	Cell adhesion, ECM component
COL5A2	P05997	Collagen alpha-2(V) chain	3.97	2.38 × 10^−3^	Developmental processes, ECM organization
CCDC80	Q76M96	Coiled-coil domain- containing protein 80	3.94	7.96 × 10^−3^	ECM organization
VCAN	P13611	Versican core protein	3.93	3.48 × 10^−3^	Developmental processes, ECM component
CILP	O75339	Cartilage intermediate layer protein 1	3.86	1.13 × 10^−3^	TGF-β signalling

Table 2 lists the top 25 most enriched proteins in the AVj compared to the LV. FC = fold change, FDR = false discovery rate. * summary of selected biological function and/or pathways. Detailed GO-annotations and reactome pathways for all DEPs are found in Table S1.

**Table 3:** Top 25 upregulated proteins of the LV.

Gene ID	Accession	Protein Description	Log2 FC	FDR	Biological Function or Pathways *
MT-ND2	P03891	NADH-ubiquinone oxidoreductase chain 2	−2.99	2.19 × 10^−3^	Mitochondrial respiratory chain
CD300LG	Q6UXG3	CMRF35-like molecule 9	−2.76	4.04 × 10^−3^	Immune system
SLC2A4	P14672	Solute carrier family 2, facilitated glucose transporter member 4	−2.24	7.20 × 10^−3^	Glucose homeostasis, response to hypoxia
SDHD	O14521	Succinate dehydrogenase [ubiquinone] cytochrome b small subunit, mitochondrial	−2.12	1.40 × 10^−3^	Mitochondrial electron transport, TCA cycle, response to hypoxia
FHL2	Q14192	Four and a half LIM domains protein 2	−2.09	1.70 × 10^−3^	Ventricular cardiac muscle, cell development
PDE1C	Q14123	Dual specificity calcium/calmodulin-dependent 3′,5′-cyclic nucleotide phosphodiesterase 1C	−2.05	3.76 × 10^−3^	Signal transduction
SDHC	Q99643	Succinate dehydrogenase cytochrome b560 subunit, mitochondrial	−2.01	1.48 × 10^−3^	Mitochondrial electron transport, TCA cycle
ANKRD2	Q9GZV1	Ankyrin repeat domain-containing protein 2	−1.94	1.36 × 10^−2^	Muscle contraction, muscle development
MYL1	P05976	Myosin light chain 1/3, skeletal muscle isoform	−1.92	1.61 × 10^−2^	Cardiac muscle contraction
MYL3	P08590	Myosin light chain 3	−1.90	4.78 × 10^−3^	Cardiac muscle contraction
ATP5MJ	P56378	ATP synthase subunit ATP5MJ, mitochondrial	−1.90	1.65 × 10^−3^	ATP synthesis
CA4	P22748	Carbonic anhydrase 4	−1.73	1.77 × 10^−3^	Metabolic process
ACSS1	Q9NUB1	Acetyl-coenzyme A synthetase 2-like, mitochondrial	−1.70	1.83 × 10^−3^	Acetyl-CoA synthesis
IDH2	P48735	Isocitrate dehydrogenase [NADP], mitochondrial	−1.69	1.41 × 10^−3^	Metabolic process, TCA cycle
MT-ATP6	P00846	ATP synthase subunit a	−1.69	1.13 × 10^−3^	ATP synthesis, response to hyperoxia
FAM210A	Q96ND0	Protein FAM210A	−1.68	1.37 × 10^−3^	Mitochondrial homeostasis, cardiac muscle contraction
TUBA8	Q9NY65	Tubulin alpha-8 chain	−1.68	2.88 × 10^−3^	Microtubule organization
C4orf54	D6RIA3	Uncharacterized protein C4orf54	−1.64	2.36 × 10^−2^	
MICOS10	Q5TGZ0	MICOS complex subunit MIC10	−1.62	2.68 × 10^−3^	Mitochondrial membrane organization
BNIP3	Q12983	BCL2/adenovirus E1B 19 kDa protein-interacting protein 3	−1.62	3.95 × 10^−3^	Mitochondrial membrane potential, apoptosis
MLYCD	O95822	Malonyl-CoA decarboxylase, mitochondrial	−1.60	1.54 × 10^−3^	Acetyl-CoA synthesis, fatty acid oxidation
PROB1	E7EW31	Proline-rich basic protein 1	−1.59	3.55 × 10^−3^	Cardiac muscle contraction
MT-CO1	P00395	Cytochrome c oxidase subunit 1	−1.59	1.25 × 10^−3^	Mitochondrial electron transport
MT-ND5	P03915	NADH-ubiquinone oxidoreductase chain 5	−1.58	1.63 × 10^−3^	Mitochondrial electron transport, response to hypoxia
SLC25A4	P12235	ADP/ATP translocase 1	−1.57	1.59 × 10^−3^	Mitochondrial transport

Table 3 lists the top 25 most enriched proteins in the LV compared to the AVj. FC = fold change, FDR = false discovery rate. * summary of selected biological function and/or pathways. Detailed GO-annotations and reactome pathways for all DEPs are found in Table S1.

**Table 4:** Significantly altered genes in the AVj compared to the LV that overlap with significantly changed proteins.

Gene ID	Accession	Protein Description	FC	Log2 FC	FDR
COMP	P49747	Cartilage oligomeric matrix protein	115	4.2	0.001
PLA2G2A	P14555	Phospholipase A2, membrane-associated	12	3.6	0.04
FMOD	Q06828	Fibromodulin	11	3.5	0.02
PRELP	P51888	Prolargin	8	3.0	0.02
CFH	P08603	Complement factor H	6	2.6	0.04
AEBP1	Q8IUX7	Adipocyte enhancer-binding protein 1	5	2.3	0.04
COL14A1	Q05707	Collagen alpha-1(XIV) chain	5	2.3	0.04
FBLN1	P23142	Fibulin-1	5	2.3	0.03
LTBP2	Q14767	Latent-transforming growth factor beta-binding protein 2	2	1.0	0.03
SERPINF1	P36955	Pigment epithelium-derived factor	2	1.0	0.04
S100A6	P06703	Protein S100-A6	2	1.0	0.03
MMP2	P08253	72 kDa type IV collagenase	2	1.0	0.04
GSN	P06396	Gelsolin	2	1.0	0.04
DCTN4	Q9UJW0	Dynactin subunit 4	0.8	−0.3	0.04
PPP6C	O00743	Serine/threonine-protein phosphatase 6 catalytic subunit	0.7	−0.5	0.02
ABRAXAS2	Q15018	BRISC complex subunit Abraxas 2	0.7	−0.5	0.03
PRKAR2A	P13861	cAMP-dependent protein kinase type II-alpha regulatory subunit	0.7	−0.5	0.04
USP10	Q14694	Ubiquitin carboxyl-terminal hydrolase 10	0.7	−0.5	0.03
SCN5A	Q14524	Sodium channel protein type 5 subunit alpha	0.6	−0.7	0.04
NCEH1	Q6PIU2	Neutral cholesterol ester hydrolase 1	0.5	−1.0	0.04

Table 4 lists the top 20 most overlapping genes/proteins between the proteomics analysis and mRNA sequencing. FC = fold change, FDR = false discovery rate.

## Data Availability

The original contributions presented in this study are included in this article/Supplementary Materials. Further inquiries can be directed to the corresponding author.

## Supplementary Materials

The following supporting information can be downloaded at https://www.mdpi.com/article/10.3390/cells13242048/s1. Table S1: Supplementary Table S1 includes statistics, MS-data as number of peptides and PSM (peptide spectra matches) and GO-terms/pathways for all significant proteins. Cut-off set to FC Log2 ±0.58 and FDR 0.05.
